# *GIDVis*: a comprehensive software tool for geometry-independent grazing-incidence X-ray diffraction data analysis and pole-figure calculations

**DOI:** 10.1107/S1600576719004485

**Published:** 2019-05-31

**Authors:** Benedikt Schrode, Stefan Pachmajer, Michael Dohr, Christian Röthel, Jari Domke, Torsten Fritz, Roland Resel, Oliver Werzer

**Affiliations:** a Institute of Solid State Physics, Graz University of Technology, Petersgasse 16, Graz 8010, Austria; b Institute of Pharmaceutical Sciences, Department of Pharmaceutical Technology, University of Graz, Universitätsplatz 1, Graz 8010, Austria; c Institute of Solid State Physics, Friedrich Schiller University Jena, Helmholtzweg 5, Jena 07743, Germany

**Keywords:** grazing-incidence X-ray diffraction, thin films, pole figures, epitaxy, computer programs, *GIDVis*

## Abstract

*GIDVis* is a software package based on MATLAB specialized for, but not limited to, the visualization and analysis of grazing-incidence thin-film X-ray diffraction data obtained during sample rotation around the surface normal. *GIDVis* allows the user to perform detector calibration, data stitching, intensity corrections, standard data evaluation (*e.g.* cuts and integrations along specific reciprocal-space directions), crystal phase analysis *etc*. To take full advantage of the measured data in the case of sample rotation, pole figures can easily be calculated from the experimental data for any value of the scattering angle covered. As an example, *GIDVis* is applied to phase analysis and the evaluation of the epitaxial alignment of pentacene­quinone crystallites on a single-crystalline Au(111) surface.

## Abstract 2

*GIDVis* is a software package based on MATLAB which is specialized for the visualization and analysis of grazing-incidence thin-film X-ray diffraction data obtained during sample rotation around the surface normal. Using *GIDVis*, detector calibration, data stitching, intensity corrections, cuts and integrations, crystal phase analysis, and calculation of pole figures can be easily performed.

## 1. Introduction   

**Figure 1:**
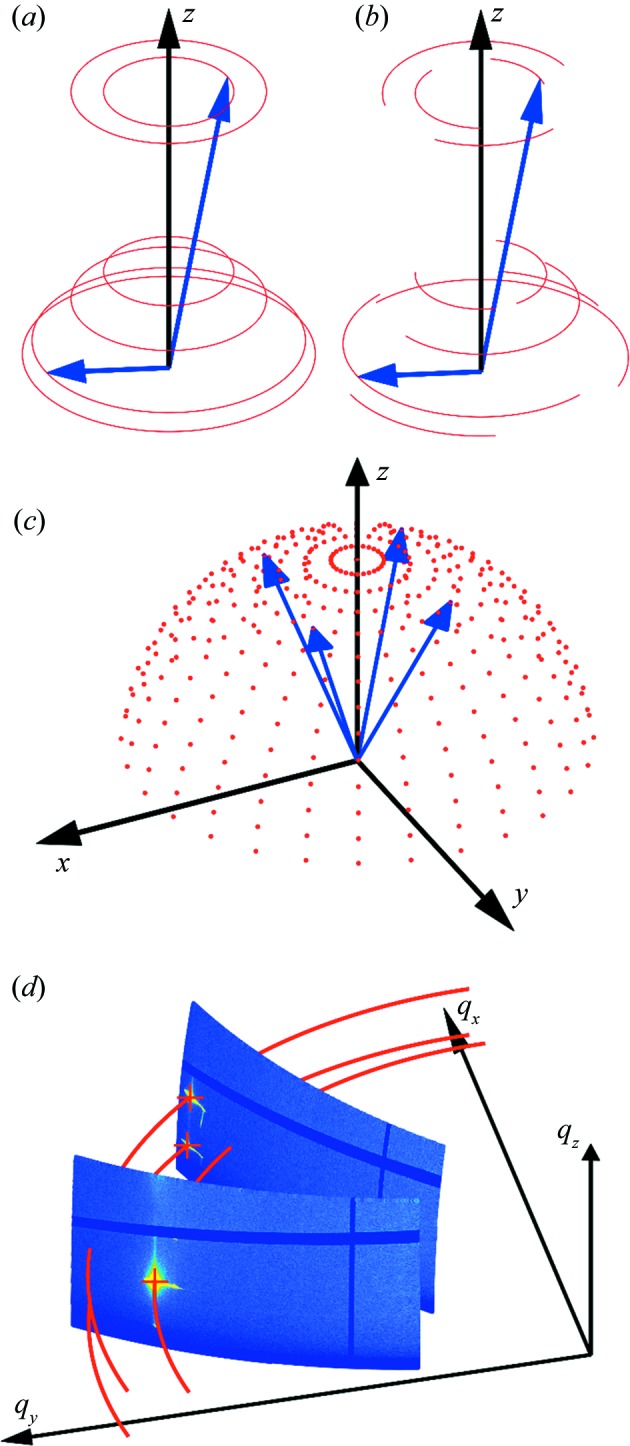
The distribution of reciprocal-lattice points (red) for samples with fibre-textured crystallites, (*a*) with the *z* axis as the rotation axis, (*b*) for samples with fibre texture combined with a partial in-plane texture, and (*c*) for azimuthally oriented crystallites. Blue arrows are selected reciprocal-lattice vectors. (*d*) Two cuts through reciprocal space by two GIXD measurements taken at different sample azimuths.

The experimental method of grazing-incidence X-ray diffraction (GIXD) has achieved huge success in the characterization of thin films and surfaces (Robinson & Tweet, 1992 ▸). The possibility of choosing an incidence angle for the primary beam close to the critical angle of total external reflection provides a number of advantages for thin-film characterization: the penetration depth into the sample system can be adjusted and the scattered intensity from the sample is enhanced considerably (Als-Nielsen & McMorrow, 2011 ▸). Several possibilities for the collection of GIXD data from films have to be considered, which are related to the texture of the crystallites within the sample (see Fig. 1 ▸). For fibre texture of crystallites or samples with random in-plane orientation of the crystallites (often found in organic thin films deposited on isotropic surfaces), GIXD studies are typically performed on static samples, *i.e.* without changing the azimuth of the sample. In these cases, the reciprocal information is distributed along rings [Fig. 1 ▸(*a*)]. One measurement at a single sample orientation, representing a cut through reciprocal space, is thus sufficient to gain access to the diffraction data for full sample analysis. However, there are several situations where the distribution of reciprocal-lattice points is not constant along rings in reciprocal space. Such cases are present in samples with large individual crystals hosted at surfaces, thus resulting in poor statistics [Fig. 1 ▸(*b*)], or epitaxially grown crystallites with a defined in-plane alignment (Haber *et al.*, 2005 ▸; Otto *et al.*, 2018 ▸) [Fig. 1 ▸(*c*)]. For both cases, the combination of a GIXD experiment with rotation of the sample is required to collect all necessary information for phase and/or texture analysis (Röthel *et al.*, 2015 ▸, 2017 ▸). Moreover, sample rotation opens new possibilities for characterization methods that are inaccessible in a simple static experiment, like the determination of in-plane mosaicity.

There are various possibilities for rotating GIXD measurements, *i.e.* several different diffraction geometries are available (*e.g.* 2 + 2, *z* axis, κ geometry *etc*; Moser, 2012 ▸; Kriegner *et al.*, 2013 ▸), allowing the measurement of diffraction data with respect to the sample surface. Irrespective of the experimental setup, the sample needs to be aligned with the incident X-ray beam. First, the sample requires precise spatial alignment (*xy* for the sample at the goniometer centre, and *z* for its height) as only this ensures that the centre of rotation is in the sample surface over the course of the experiments. Then the incident angle is set, typically in the range of the critical angle α_c_ (the angle below which total external reflection occurs) up to few degrees. Higher incident angles allow for a reduction in the beam footprint on the sample surface, which is crucial in terms of in-plane smearing and *q_z_* resolution when using two-dimensional detectors [*q* = (4π/λ)sinθ, where θ is half the scattering angle and λ is the wavelength of the incident radiation].

After the alignment process, the scattering information for the first azimuthal position is collected, followed by sample rotation around the surface normal and another image being taken [*cf*. Fig. 1 ▸(*d*)]. This is repeated until the entire upper hemisphere (Φ = 0–360°) is mapped. It should be noted that the incident angle has to be the same for each sample position. Considering the different geometries, this is achievable either by a complex and time-consuming adjustment of various moveable parts (goniometer and motor positions) at each point or by proper design of the sample movements, as for example offered by the κ or Eulerian geometry, which directly allow sample rotation around the surface normal. The data quality improves further if the intensity is collected continuously during azimuthal rotation as opposed to a stepped scan, so that information, even though smeared because of integration, is fully collected.

Diffracted intensities can be collected by various detectors. The current state of the art are solid-state area detectors which provide information on a large angular range together with fast data acquisition. The drawback here is that, owing to construction limitations, blind areas exist on the detector. These can be readily accepted for experiments with sufficient redundant data, but otherwise additional measurements need to be taken. Hereby the detector is moved by a certain amount by the goniometer or laterally, so that the blind areas point towards other areas of reciprocal space. From these additional measurements, (larger) images containing all of the diffraction information can be obtained.

To extract reliable information from the experimental data, several data processing and evaluation steps are required. There are a number of helpful software packages which assist in the visualization and analysis of (grazing-incidence) X-ray diffraction or small-angle X-ray scattering [(GI)SAXS] data (Benecke *et al.*, 2014 ▸; Breiby *et al.*, 2008 ▸; Hammersley, 2016 ▸; Jiang, 2015 ▸; Lazzari, 2002 ▸). There is also a specific solution for data extraction from three-dimensional reciprocal-space maps (Roobol *et al.*, 2015 ▸), *e.g.* collected by GIXD from rotating samples (Mocuta *et al.*, 2013 ▸).

Although software packages specializing in SAXS [*e.g.* *DPDAK* (Benecke *et al.*, 2014 ▸) and *GIXSGUI* (Jiang, 2015 ▸)] can typically be used for GIXD data visualization and reduction, analysis of diffraction data requires other features typically not available in SAXS software, *e.g.* calculation of expected peak positions and intensities from a known crystal structure, support for detectors mounted on goniometer arms, and subsequent data stitching or, in the case of textured samples, the extraction of pole figures.

Here we present the software *GIDVis*, which is a comprehensive tool for the data analysis of GIXD data of static or rotating samples, incorporating many aspects of other tools within one program, and adding additional and easy-to-use features for the evaluation of rotating GIXD data. The software is capable of dealing with all kinds of data, including linear and area-detector data from static detectors or detectors mounted on goniometer arms. It allows the user to perform all basic data handling like summation or stitching of data from different detector positions and contains a full set of tools to perform an evaluation of crystallographic properties.

## 2. Experimental procedure and data transformation   

**Figure 2:**
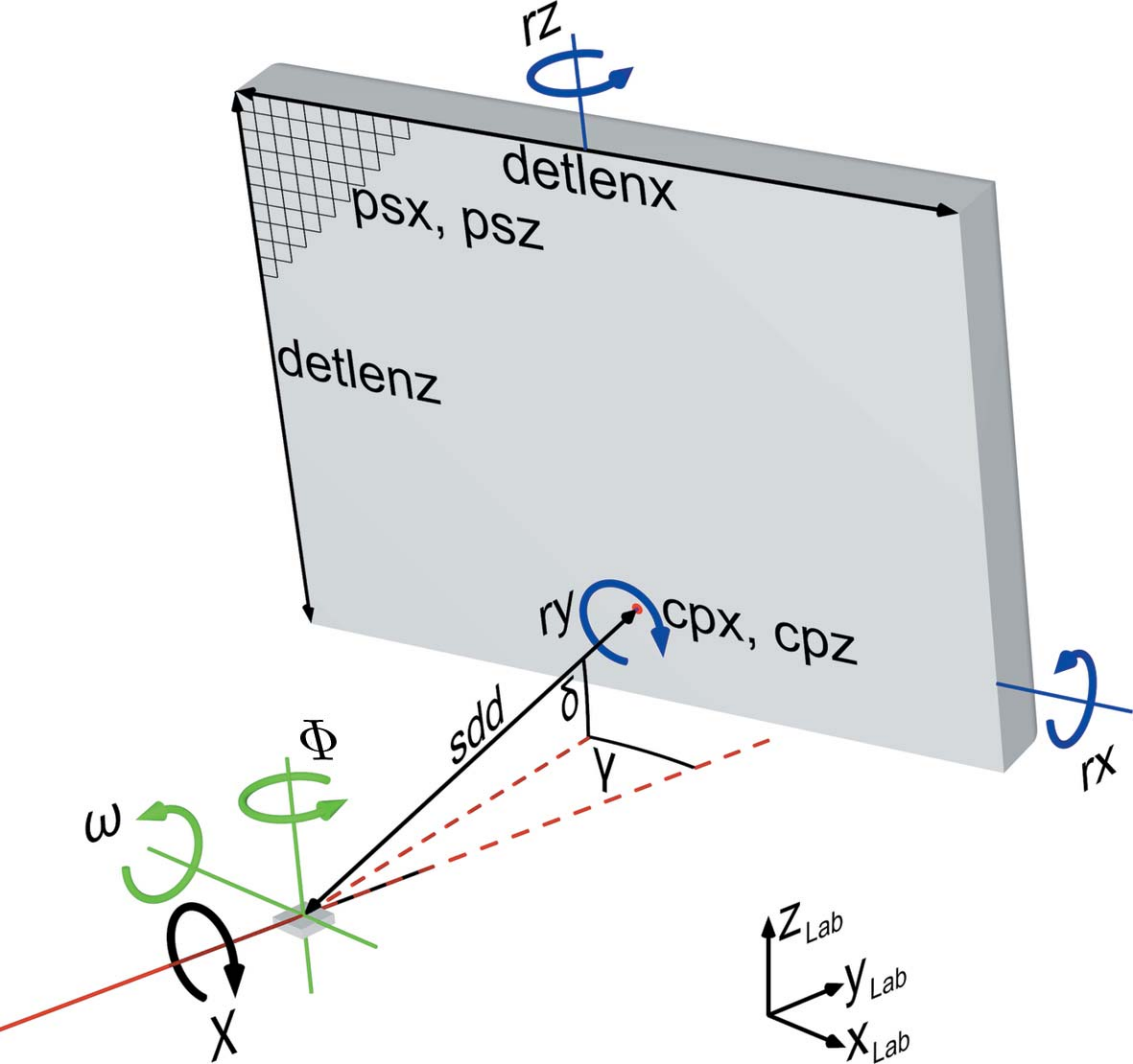
A typical measurement setup using an area detector mounted on a goniometer arm. The coordinate system describes the directions of the laboratory system. Rotations within the detector coordinate system are indicated in blue, rotations within the sample coordinate system in green, and rotations in the laboratory system in black.

*GIDVis* uses various details from the experimental setup, including angles, distances, wavelength and the pixel size of the detector, to convert the diffraction data from the pixel space of the detector into reciprocal space. A summary of the required experimental parameters is provided in Fig. 2 ▸. The detector is described by *detlenx* times *detlenz* pixels of size *psx* and *psz*. Their positions are defined by the goniometer angles δ and γ and the sample-to-detector distance sdd. Any non-orthogonality of the detector relative to the primary beam for δ = γ = 0° is described by the rotations *rx*, *ry* and *rz*. The sample position is set by the angular movements ω, χ and Φ. Additionally, the wavelength and the centre pixel position *cpx*/*cpz*, *i.e.* the pixel position of the direct beam, must be known. From these parameters one can directly calculate all necessary transformations so that finally the diffraction information is present in reciprocal-space coordinates. This has the advantage that measurements from other experimental stations or experimental setups are directly comparable without requiring knowledge about the specific setup. While such a procedure is directly accessible, inaccuracies in the angles or distances used have a large influence on the correctness and quality of the reciprocal data. Therefore, it is best practice to perform an additional detector calibration measurement beforehand. Here, *GIDVis* provides the possibility of extracting the necessary parameters using standards like lanthanum hexaboride (LaB_6_; Black *et al.*, 2010 ▸), silver behenate (Huang *et al.*, 1993 ▸), silicon standards (Black *et al.*, 2010 ▸) or custom calibrants. Based on these data, the transformation to reciprocal space is quite precise.

## 3. Pole-figure calculation   

**Figure 3:**
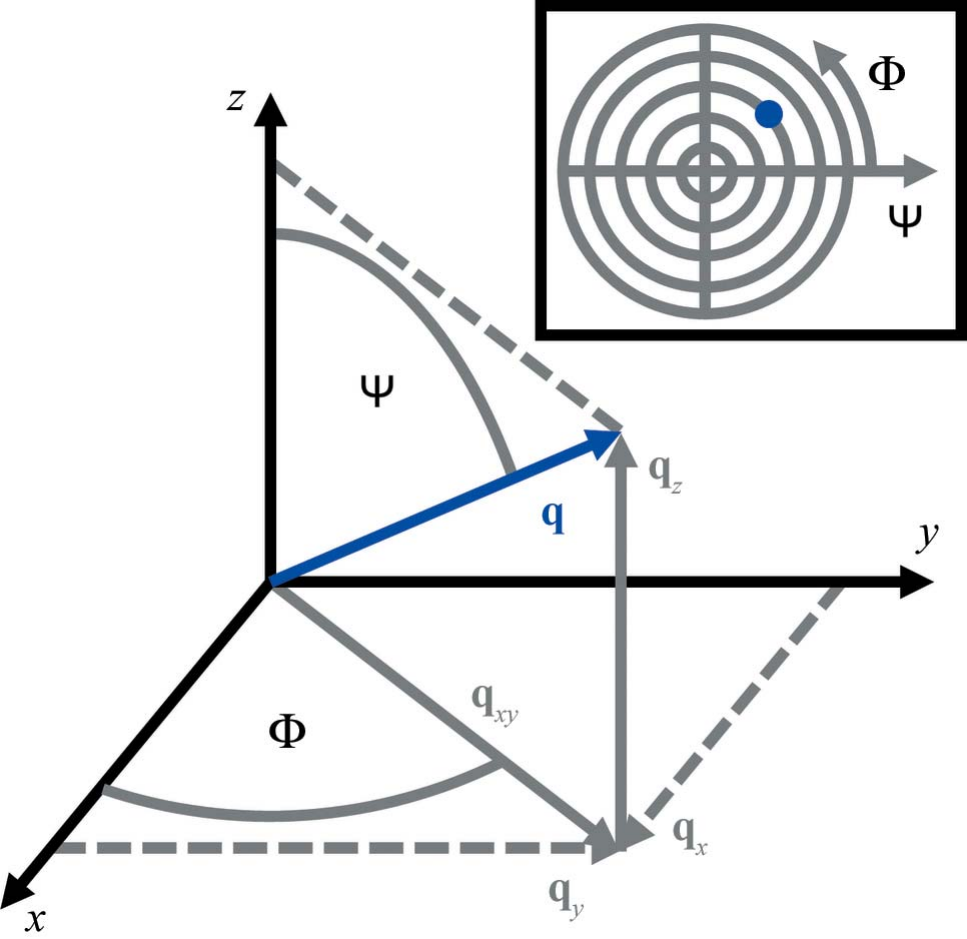
The angular relationships in the sample coordinate system used by the pole-figure calculation, and (inset) the approximate position of the plotted scattering vector **q** in the pole figure.

For some types of sample, the angle Φ is of no particular interest, so that information along *q_x_* and *q_y_* is merged into *q_xy_* = 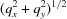, *i.e.* the component of the scattering vector parallel to the sample surface. This also means that information on the azimuth is lost. By including information from the angle Φ, reciprocal-space information in all directions, *i.e.* *q_x_*, *q_y_* and *q_z_*, can be determined. Fig. 3 ▸ shows an example of the scattering vector **q** in the sample coordinate system. The scattering vector can be separated into its in-plane component *q*_*xy*_ and the out-of-plane component *q*_*z*_. The inclination of **q** with respect to the *z* axis is described by the angle Ψ, ranging from 0 to 90°, and the angle Φ is defined as the angle between the in-plane component of the scattering vector *q*_*xy*_ and the *x* axis, going from 0 to 360°. So instead of using *q*_*xy*_ and *q*_*z*_, the direction of the scattering vector is defined by the two angles Ψ and Φ (Alexander, 1979 ▸). Following the described definitions, they can be determined by
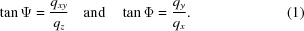



In a pole figure, the spatial distribution of the pole directions of certain net planes (defined by a distinct *q* value or a *q* range with a certain width) is plotted in a single polar plot with the radius being Ψ and the azimuthal part Φ (*cf*. Fig. 3 ▸, inset) and the measured intensity is colour coded. For practical reasons, a stereographic projection is chosen for visualization. In *GIDVis*, pole figures can be calculated from the experimental GIXD patterns and visualized directly. The data can also be converted for analysis with other software (Salzmann & Resel, 2004 ▸) to determine the epitaxial relationship between the adsorbate and substrate and to obtain the orientation distribution function (ODF) (Alexander, 1979 ▸; Suwas & Ray, 2014 ▸).

## 4. Workflow   

**Figure 4:**
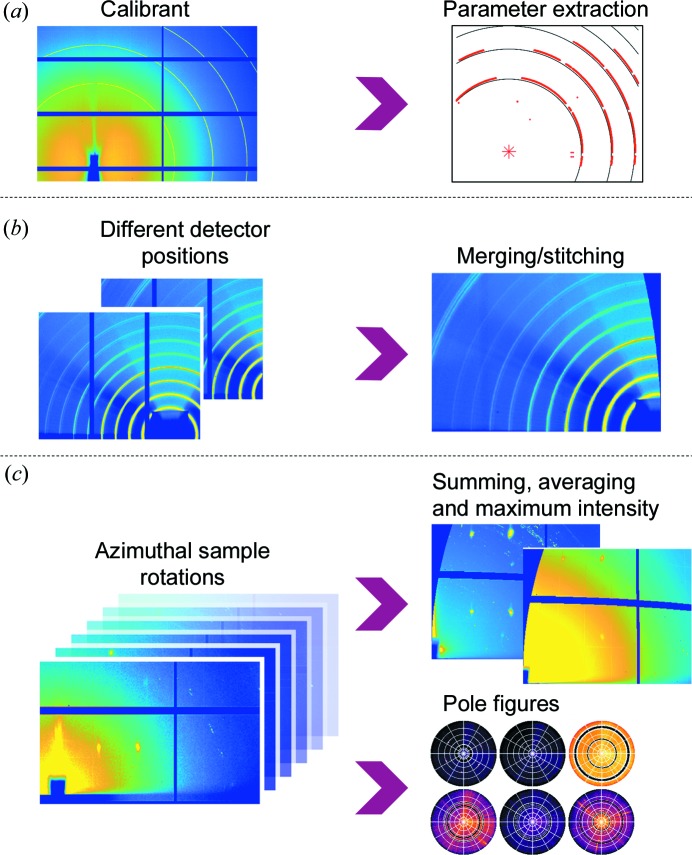
A schematic diagram of the data processing in *GIDVis*. (*a*) Starting from a standard measurement and calibration parameter extraction from it, the data (*b*) can be stitched/merged if necessary and (*c*) can be transformed to reciprocal space independently of the input and visualized in a variety of ways.

Fig. 4 ▸ shows a typical workflow employed in *GIDVis*. Starting from measurements of a polycrystalline powder calibrant with a well known interplanar spacing, the experimental parameters are extracted by comparison of the expected and actual measured peak positions [Fig. 4 ▸(*a*)]. The obtained calibration parameters are stored and can easily be applied to any other two-dimensional diffraction pattern recorded using the same setup to calculate the reciprocal-space or polar representation.

The detector gaps due to the construction restrictions of the detector leave some inaccessible areas which might cause problems. Having the possibility of recording diffraction images at different detector locations, using either a goniometer arm or a simple detector translation, the software is capable of using several data sets to generate a single merged data set without detector gaps and covering a larger volume of reciprocal space [Fig. 4 ▸(*b*)].

For a sample of poor statistics or high in-plane order, an experiment using a 360° azimuthal rotation is best. For some samples, it might be sufficient to collect all information obtained during the rotation within a single image. However, if several images at distinct azimuths are recorded, *GIDVis* allows the user to combine the full diffraction information in one image afterwards by summing, averaging and extracting the maximum intensity of each pixel during the rotation. This provides a convenient way of reducing the data for an initial texture and polymorph phase analysis. Additionally, pole figures can easily be calculated, which takes full advantage of collecting data for the entire upper hemisphere [Fig. 4 ▸(*c*)].

Independently of the input data type – static, azimuthal sample rotations, different detector positions (including merged/stitched images) – several data evaluation routines, *e.g.* cuts and integrations along specific reciprocal-space directions, crystal phase analysis, intensity corrections, fitting of peak positions, transformation to powder-like patterns *etc.*, are available. Moreover, *GIDVis* can easily be used directly during measurements, *e.g.* to support sample alignment by extraction of height scans and rocking curves from two-dimensional intensity data, which can be directly evaluated further for the correct sample position, similar to what is done with a point detector. Because of the real-time data conversion to reciprocal space, *GIDVis* can also be used to monitor the measurement results, *e.g.* to make decisions on the optimum incident angle.

*GIDVis* is engineered using a very modular structure, allowing many different tasks to be carried out directly within a single program (automatic intensity extraction, structure data comparison or even a rudimentary structure viewer). For further demands, the modular structure means that *GIDVis* is highly adaptable and, more importantly, can be extended to even more specific needs. Interfaces to indexing routines using the diffraction pattern calculator (*DPC*; Hailey *et al.*, 2014 ▸) or *CRYSFIRE* (Shirley, 2006 ▸) might be easily generated, as well as comparisons with literature structure data (*e.g.* automatic structure searches). The model-fitting routines employing Gaussian fits implemented in *GIDVis* might be expanded using models suitable for SAXS and GISAXS (Hexemer & Müller-Buschbaum, 2015 ▸; Schwartzkopf & Roth, 2016 ▸). Other expansions could make corrections for multiple scattering effects in GIXD data available (Resel *et al.*, 2016 ▸) or handle dynamic diffraction effects in general to obtain more accurate peak intensity values and positions, as usually performed via the distorted-wave Born approximation (DWBA) (Daillant & Alba, 2000 ▸; Lazzari, 2009 ▸).

## 5. Example: pentacene­quinone on Au(111)   

To demonstrate the advantage of using rotating GIXD and *GIDVis* we provide the example of measurements of epitaxially grown pentacene­quinone (P2O) on an Au(111) single-crystalline surface. P2O (pentacene­quinone, or pentacene-6,13-dione, C_22_H_12_O_2_, CAS number 3029-32-1) is an organic semiconductor and is already known to exhibit several polymorphic phases (Simbrunner *et al.*, 2018 ▸; Salzmann *et al.*, 2011 ▸; Nam *et al.*, 2010 ▸; Dzyabchenko *et al.*, 1979 ▸).

Prior to deposition of the molecule, the substrate surface was cleaned by repeated cycles of Ar^+^ sputtering with an energy of 600 eV and thermal annealing at 773 K. The mol­ecular film was then deposited from an effusion cell at a constant temperature of 463 K under ultra-high-vacuum conditions (base pressure 10^−10^ mbar = 10^−8^ Pa) directly onto the substrate held at room temperature. The film thickness was monitored *in situ* using optical differential reflectance spectroscopy (Forker & Fritz, 2009 ▸) and was calculated to be ten (not necessarily full and densely packed) layers.

After sample transfer to ambient conditions, the sample was investigated on the XRD1 beamline at the Elettra Synchrotron, Trieste, Italy, using a wavelength of 1.40 Å and a Pilatus 2M detector approximately 200 mm away from the sample. After setup calibration using an LaB_6_ standard, sample alignment was performed using rocking curves, height scans and translation scans (*x* and *y*) to locate the midpoint of the substrate surface in the centre of rotation for all relevant movements required during rotating GIXD data collection. For all these scans, *GIDVis* was used for fast extraction of two-dimensional scans from sets of two-dimensional images and subsequent evaluation. For the rotating GIXD measurements the incident angle was set to 0.4°, which corresponds to around 80% of the critical angle of gold at this wavelength (Henke *et al.*, 1993 ▸). Using an exposure time of 10 s per image, 180 single images with a Φ step size (azimuthal rotation) of 2° were collected, so that information from a full 360° sample rotation was obtained. Diffraction data were recorded continuously, which means that even for very narrow peaks the intensity was still collected.

In the first step after the data collection, these data were transferred to reciprocal space. Inspection of individual images revealed that the single images do not look identical (data not shown). From this initial information it can be directly concluded that there is some in-plane texture, as expected for an epitaxially grown film.

**Figure 5:**
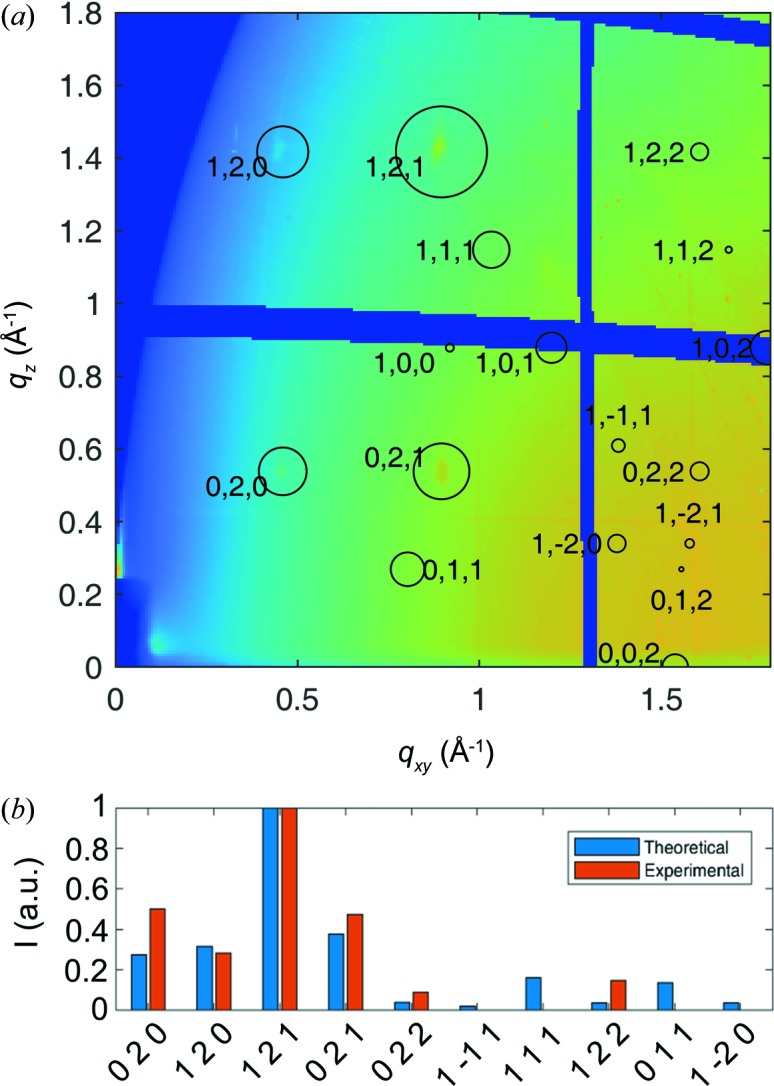
(*a*) The summation of intensity from all 360° azimuthal directions, and (*b*) a comparison of the expected and measured intensities.

In the next step, one can sum the intensities of all images pixel by pixel to construct an integrated image [Fig. 5 ▸(*a*)]. Such data then allow the identification of the contact plane, *i.e.* the crystallographic plane parallel to the substrate surface, and the polymorphic phase. A comparison of the measured peak positions with the expected positions from literature crystal structure solutions reveals the presence of the crystal phase reported by Dzyabchenko *et al.* (1979 ▸) with a (140) contact plane. *GIDVis* can directly plot the expected peak positions (centres of the rings) together with the squared absolute values of the structure factors (proportional to the areas of the rings) (*cf*. Fig. 5 ▸). Note that the measured intensity is corrected in terms of geometric factors, *i.e.* Lorentz and polarization factor, solid angle, pixel distance and detector efficiency corrections are applied. Using *GIDVis*, extracting the intensities by fitting a two-dimensional Gaussian function with a background plane is easily done and allows us to compare the measured intensity with the available structure solution using other representations [*cf*. Fig. 5 ▸(*b*)]. The intensities of most of the peaks are reproduced with good agreement, except for the 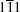, 111, 011 and 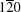 peaks where no intensity could be extracted, *i.e.* the fit did not return a result. Note that the expected intensities of these peaks are very small.

To study the in-plane alignment of the crystallites in more detail, pole figures can easily be generated within *GIDVis*. Here, there are some advantages of the described approach compared with laboratory-based pole-figure investigations. The first is the greatly reduced measurement time, which is here about 30 min for a large range of *q* values compared with at least 15 h for a single *q* value using in-house texture goniometers. From 180 GIXD measurements of a 360° sample rotation, pole figures can be obtained for any value of the scattering angle covered in the images by extracting it from the present data set. This limitation is often reflected in the fact that reciprocal-space mapping, although a very powerful technique, is in fact rarely done in the home laboratory (Resel *et al.*, 2007 ▸), and usually epitaxy is tested for known phases only. The use of synchrotron radiation and the approach we implement makes the calculation of pole figures and their inspection for materials of unknown crystal structure reasonable and thus possible. Because of the geometry chosen in GIXD, even pole figures with very low *q* values can be calculated, which are often hard to access with classical texture goniometers owing to the strong background of the primary beam. Besides these advantages, elimination of the blind areas of the area detector would require three measurements with slightly different detector positions. Yet, as the information is often redundant because of higher-order reflections or scattering into another quadrant of a large detector, this is of minor importance for this kind of experiment. A real limitation of the GIXD geometry is its inherent insufficiency for measuring close to the specular direction (*i.e.* low *q_x_* and *q_y_* and thus *q_xy_* values) due to the external reflection of the beam. Here, classical pole-figure geometries (*e.g.* Eulerian cradles) would be required, but at the expense of lacking the advantages of GIXD measurements.

**Figure 6:**
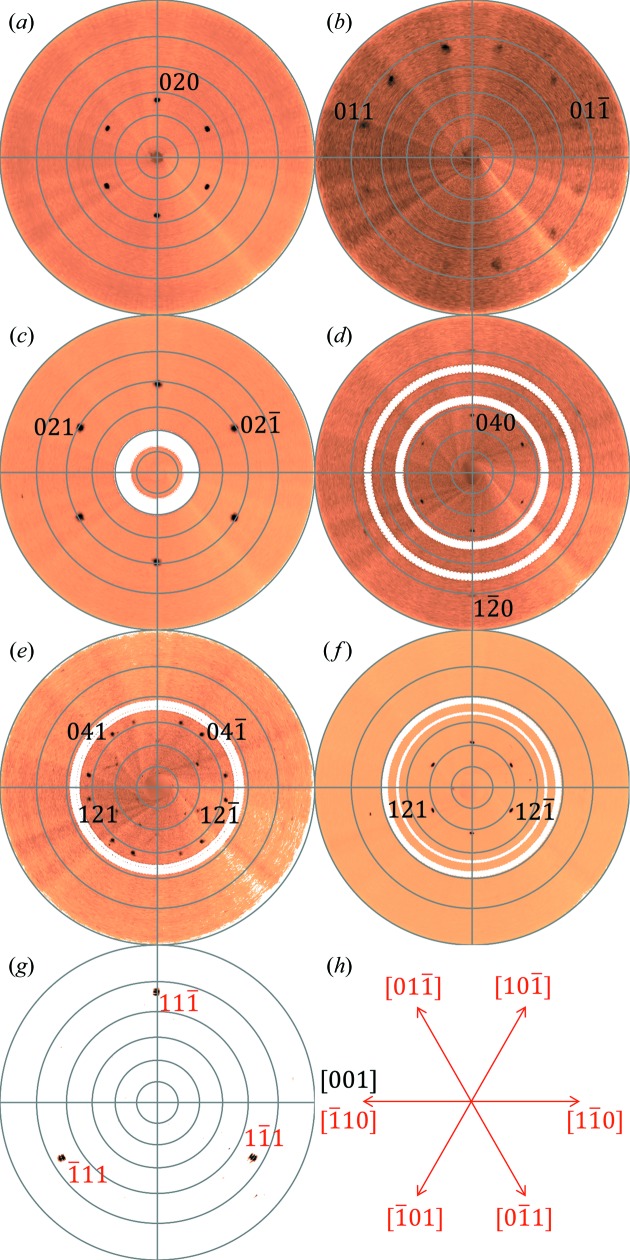
(*a*)–(*g*) A series of relevant pole figures at distinct *q* values, indexed with the bulk crystal structure of pentacene­quinone in (140) orientation (black) and gold in (111) orientation (red). (*h*) The crystal directions in real space of the substrate (red) and the organic overlayer (black).

Fig. 6 ▸ shows several pole figures calculated from the rotating GIXD experiment, allowing the determination of the in-plane orientation of the P2O crystallites with respect to the single-crystalline Au(111) substrate. For detailed analysis, the pole figures were exported from *GIDVis* as .rwa files and analysed using the standalone software *Stereopole* (Salzmann & Resel, 2004 ▸). Missing data points due to detector gaps are present as white concentric circles in Figs. 6 ▸(*c*)–6 ▸(*f*). For several of the pole figures, six areas of high intensity (enhanced pole densities, EPD) are found [Figs. 6 ▸(*a*), 6 ▸(*c*) and 6 ▸(*f*)], while there is also one with only three [Fig. 6 ▸(*g*)]. The others show even more EPD within a single pole figure [Fig. 6 ▸(*b*), 6 ▸(*d*) and 6 ▸(*e*)]. Using the information gained from the integral measurements, we already know that the EPD can be explained by the P2O bulk crystal structure in a (140) orientation. Together with the sixfold gold surface symmetry, all of the observed peaks can be explained. Note that both the reciprocal-space map and the pole figures could also be explained with the crystallographic equivalent orientation 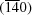.

A pole figure of the single-crystalline Au(111) substrate allows the determination of the symmetry directions of the gold surface [*cf*. Fig. 6 ▸(*g*)]. These crystallographic directions are indicated by arrows in an orientation image [Fig. 6 ▸(*h*)]. Using the same approach for the organic layers and comparing the results with those of gold shows that the main axis in plane, *i.e.* [001], is aligned along the gold 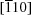 axis. To summarize, the following relationships between the substrate and the organic layer are found: (111)_Au_ || ±(140)_P2O_ and 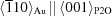.

## 6. Availability   

*GIDVis* is based on MATLAB and released under the terms of the GNU General Public Licence, either version 3 of the licence or any later version. It can be obtained at https://www.if.tugraz.at/amd/GIDVis/ free of charge. Two download options are provided. (i) For users without MATLAB, executable files for Windows and Linux are provided. To run, they require the MATLAB runtime, which can be downloaded from The Mathworks Inc. (https://mathworks.com/products/compiler/matlab-runtime.html) free of charge. (ii) The *GIDVis* source code is also provided in our online repository, allowing users to adapt the program to their needs (requires MATLAB).

Extended tutorials, additional help and the theoretical background of the algorithms implemented can be found in a separate documentation file that can also be downloaded from the web page mentioned above.
